# Prognostic and predictive value of IDO expression in metastatic melanoma treated with Ipilimumab

**DOI:** 10.1371/journal.pone.0321937

**Published:** 2025-05-07

**Authors:** Dagmar von Bubnoff, Christina Schmitt, Simone M. Goldinger, Dirk Schadendorf, Katharina C. Kähler, Christian Hafner, Nora Kramer, Waltraud Fröhlich, Reinhard Dummer, Carola Berking, Stefan Schliep, Michael C. Kirchberger, Lucie Heinzerling

**Affiliations:** 1 Department of Dermatology, University Hospital Lübeck, Lübeck, Germany; 2 Department of Dermatology and Allergy, University Hospital, LMU Munich, Munich, Germany; 3 Department of Dermatology, University Hospital of Zurich, Zürich, Switzerland; 4 Department of Dermatology, University Hospital Essen, Essen, Germany; 5 Department of Dermatology, University Hospital Schleswig-Holstein, Campus Kiel, Kiel, Germany; 6 Department of Dermatology, University Hospital Regensburg, Regensburg, Germany; 7 Department of Dermatology, Universitätsklinikum Erlangen and Friedrich-Alexander-Universität (FAU) Erlangen-Nürnberg, Erlangen, Germany; 8 Comprehensive Cancer Center Erlangen-European Metropolitan Area of Nuremberg (CCC ER-EMN), Erlangen, Germany; 9 Deutsches Zentrum Immuntherapie, Erlangen, Germany; Bowen University, NIGERIA

## Abstract

**Background:**

The tumor microenvironment is crucial for prognosis and response to immunotherapy in several tumor entities.

**Methods:**

In a multicenter retrospective study, a total of 86 tumor samples from patients with metastatic melanoma were evaluated for baseline expression of indoleamine 2,3-dioxygenase (IDO) and programmed death ligand 1 (PD-L1). Expression patterns of IDO and PD-L1 on tumor cells and antigen-presenting cells (APCs) as determined by immunohistochemical (IHC) staining of paraffin-embedded tissue sections were correlated with response to ipilimumab and overall survival (OS). Statistical analysis was performed using the Spearman correlation, the Mann-Whitney test and Kaplan-Meier estimator.

**Results:**

IDO expression in tumor cells or APCs was not predictive for treatment response. The median OS was 26 months in IDO-positive and IDO-negative patients, regardless of IDO expression in tumor cells or APCs. A correlation of IHC expression scores of IDO and PD-L1 could not be documented.

**Conclusion:**

The exact role of IDO in creating an immunosuppressive tumor environment and its reversal needs to be further elucidated.

## Introduction

The tumor microenvironment (TME) is crucial for prognosis and response to immunotherapy [1,2]. To identify potential responders and avoid unnecessary morbidity due to side effects [3,4], investigations for predictive biomarkers are ongoing [5–9]. PD-L1 determines response to immunotherapy in lung cancer and to a lesser extent in melanoma [10,11] since it influences immune tolerance [12,13] and can be blocked by anti-programmed cell death protein 1/programmed death ligand 1 (PD-1/PD-L1) inhibitors [3]. Checkpoint inhibiting antibodies (ICI) targeting cytotoxic T-lymphocyte antigen 4 (CTLA-4) and PD-1 are a major breakthrough in cancer treatment [14,15].

Indoleamine 2,3-dioxygenase (IDO) has gained interest as a target in cancer therapy since it also contributes to an immunosuppressive tumor environment. Furthermore, IDO expression in primary cutaneous tumors correlates with progression-free survival [16]. IDO plays an important role in immune regulation by metabolism of the amino acid tryptophan to kynurenine; increased kynurenine in the tumor microenvironment can decrease CD8 + T cells and natural killer cells, and increase regulatory T cells and myeloid-derived suppressor cells [17,18]. IDO is an intracellular enzyme comprising heme and is expressed in a variety of immune and stromal cells including plasmacytoid dendritic cells and monocytic myeloid-derived suppressor cells [16,19]. It regulates fetal rejection by maternal T lymphocytes and autoimmune processes like graft-versus-host disease (GVHD) [20]. In cancer, IDO has been demonstrated to block effective antitumor responses [21–24] by, e.g., arresting T-lymphocytes in G1 phase, promoting T-cell and dendritic cell apoptosis, and supporting regulatory T-cell generation [25–29]. Furthermore, tryptophan metabolites inhibit NK-cell function [30,31]. IDO expression has been shown to be regulated by interferon-gamma. Thus, tumors with T-cell infiltrates are more likely to show enhanced IDO expression [21,22,24]. It is also overexpressed in some tumor types including breast, ovarian, hepatocellular, lung, gastric and colorectal cancers, where higher expression has been associated with worse prognosis. In melanoma, peritumoral expression of IDO in primary tumors has been associated with poor prognosis [19].

Consequently, IDO inhibitors have been evaluated as therapeutic drugs in melanoma and other cancer entities. Despite the promise, first clinical studies showed no effect as monotherapy in advanced solid cancers including melanoma or colorectal carcinoma, using the orally applied IDO inhibitors epacadostat [32] or indoximod [33]. Murine studies demonstrated that CTLA-4 blockade strongly synergizes with IDO inhibitors and thus lead to the design of clinical trials [24]. In a phase 1/2 study, the combination of the orally applied IDO inhibitor epacadostat with intravenous ipilimumab demonstrated clinical activity with objective response rates of 26% in immunotherapy-naïve melanoma patients [34]. As a follow-up, IDO inhibitors were tested in combination with anti-PD1 and anti-PD-L1 inhibitors [35,36]. The promising results of early trials of IDO inhibitors in metastatic melanoma [19,34,36,37] however, were tempered by a negative phase III trial [38]. In this study, response rates were similar between intravenous anti-PD-1 therapy only and anti-PD1 in combination with IDO inhibitor, with an objective response rate of 32% and 34%, respectively [38]. Furthermore, progression-free survival did not differ significantly between treatment groups [38].

In contrast, a phase 1/2 trial recently investigated a new treatment approach in metastatic melanoma patients consisting of a subcutaneously injected vaccine against IDO and PD-L1 in combination with nivolumab. Here, an objective response rate of 80% (CI, 62.7–90.5%) and a median progression-free survival of 26 months (CI, 15.4–69 months) was demonstrated [39]. This vaccination approach could show the modulation of the tumor microenvironment through a localized activation of TH1-cells leading to a localized inflammation in tumor tissue, changing the TME into a ‘hot’ one [39]. Preceding the clinical trials, a mouse model with class I MHC directed vaccines against IDO peptide had elucidated this mechanism [40].

These findings as well as data from metabolomics studies [41,42] underline the importance of understanding the role of IDO in combination with PD-L1 for tumor response and survival of cancer patients. Thus, we investigated the influence of expression patterns of IDO particularly in melanoma metastases, its correlation to PD-L1 expression and its association with response to checkpoint inhibitor therapy as well as the prognostic value regarding overall survival.

## Materials and methods

### Study design

This multicenter retrospective study included patients from 6 skin cancer centers (University Hospitals Erlangen, Tübingen, Zürich, Kiel, Essen, and Regensburg) after obtaining approval from the local ethics committee of the FAU University Hospital Erlangen-Nürnberg (No. 261_13B). All centers were acting according to their regulatory requirements. Patients gave written informed consent before inclusion. Data was accessed in December 2017, individuals could not be identified during analyses and evaluations. The study included patients with metastatic melanoma who had a baseline tumor biopsy before start of checkpoint inhibitor therapy.

Patients had received ipilimumab according to study protocol or standard prescription guidelines with predominantly 4 doses of 3 mg/kg IV every 3 weeks (S1 Table). The cohort was enriched for patients with a response to ipilimumab.

### Tumor specimen

Pretreatment tumor samples from resected metastases (including cutaneous, subcutaneous, lymph node, visceral, and brain metastases) or primary tumors were assessed for IDO and PD-L1 expression. Tissue samples were obtained before initiation of ipilimumab treatment. No cytology specimens or core biopsies were included. All specimen were reviewed histologically for tumor content and immunohistochemically stained for different melanoma markers including HMB45, S100, SOX10, Melan A and Mage A3. In addition, tumor content was quantitatively assessed. For analyses, only slides with at least 5% tumor content were included.

### Immunohistochemical staining

In total, 86 biopsies were included for immunohistochemical analysis of IDO and PD-L1 expression. Staining was performed using the anti-IDO monoclonal antibody (dilution 1:200) from Millipore^TM^, Billerica, USA (clone 10.1) and anti-PD-L1 monoclonal antibody (dilution 1:100–1:400) from Cell Signaling Technology®, Massachusetts, USA (clone E1L3N).

Anti-IDO antibody, clone 10.1, was chosen since it has been widely used to detect IDO in immunohistochemistry (IHC) [43–46] and was successfully tested in experiments with several IDO-antibodies on different tissues including positive controls, i.e., palatine tonsil. Clone 10.1 gave consistent coherent results. For PD-L1 (E1L3N), newer publications have shown E1L3N as a consistently reliable antibody with comparable performance to other clones [47–49]. This is in accordance with our experience, since in our clinic it was used in routine IHC staining.

Since exclusion of one of several tissue samples of the same patient would have added bias, for some patients several samples, i.e., skin and nodal biopsies, were included in the analysis.

An appropriate isotype-matched antibody was used as control. The IHC scoring included (-), (+), (++) and (+++), also described as 0, 1, 2 and 3, evaluated in ten representative high-power fields. Score intensity (- or 0) was defined as negative, (+ or 1) was defined as < 25% of IHC positive cells, (++ or 2) included 25–50% of IHC positive cells, whereas more than 50% of positive cells were defined as (+++ or 3). For IDO, assessment of slides was performed by 2 independent reviewers scoring from (-) to (+++) in two compartments, tumor cells and antigen presenting cells (APCs). For identification of APCs, the anti-IDO antibody (clone 10.1) was combined with double-immunofluorescence staining against CD11c (5D11, dilution 1:50, Novocastra) and CD68 (PGM1, dilution 1:50, Novocastra).

Cells were scored as IDO-negative (IDO-) or IDO-positive if < 25% (IDO+), 25–50% (IDO++), or > 50% (IDO+++) of melanoma cells or APCs were IDO-positive. For PD-L1, assessment was also conducted by independent reviewers from (-) to (+++) in tumor tissue samples, examples for IHC intensity scoring are demonstrated in Fig 4A–D. The reviewers were blinded for the clinical outcomes.

**Fig 1:**
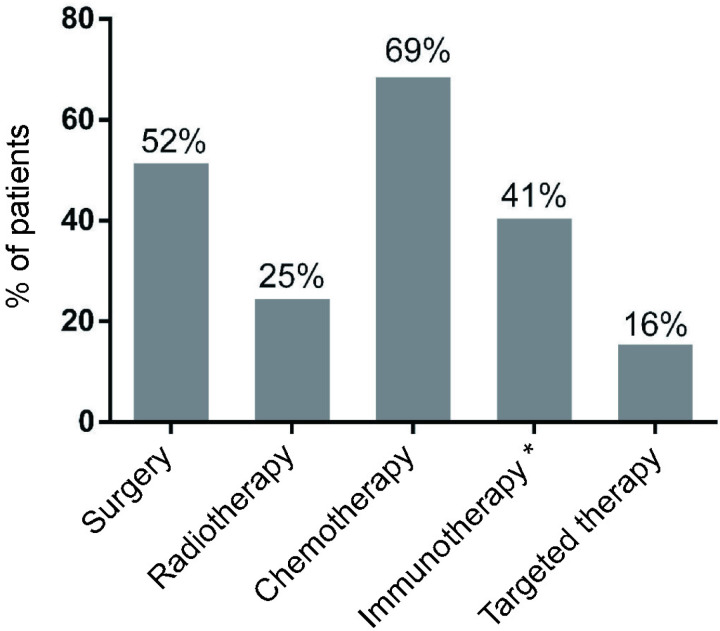
Pretreatment modalities of patients before treatment with ipilimumab (n = 86). *Interferon alpha, IL-2, other studies or dendritic cell vaccination.

**Fig 2:**
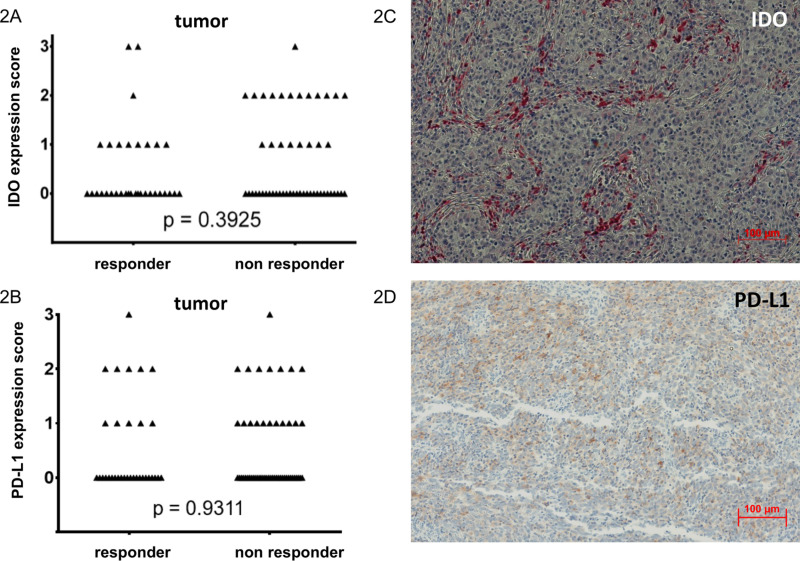
IDO and PD-L1 expression in ICI responders and non-responders. (A) IDO expression and (B) PD-L1 expression in tumor cells of patients with therapy benefit (CR, PR or SD), so-called ‘responders’, and non-responding patients (PD) to therapy with ipilimumab. n = 86. PD: progressive disease; SD: stable disease; PR: partial response; CR: complete response. IHC staining in metastasis. Performed with a monoclonal antibody against IDO (C) and against PD-L1 (clone E1L3N) (D). Hundredfold magnification. ICI: immune checkpoint inhibition.

**Fig 3:**
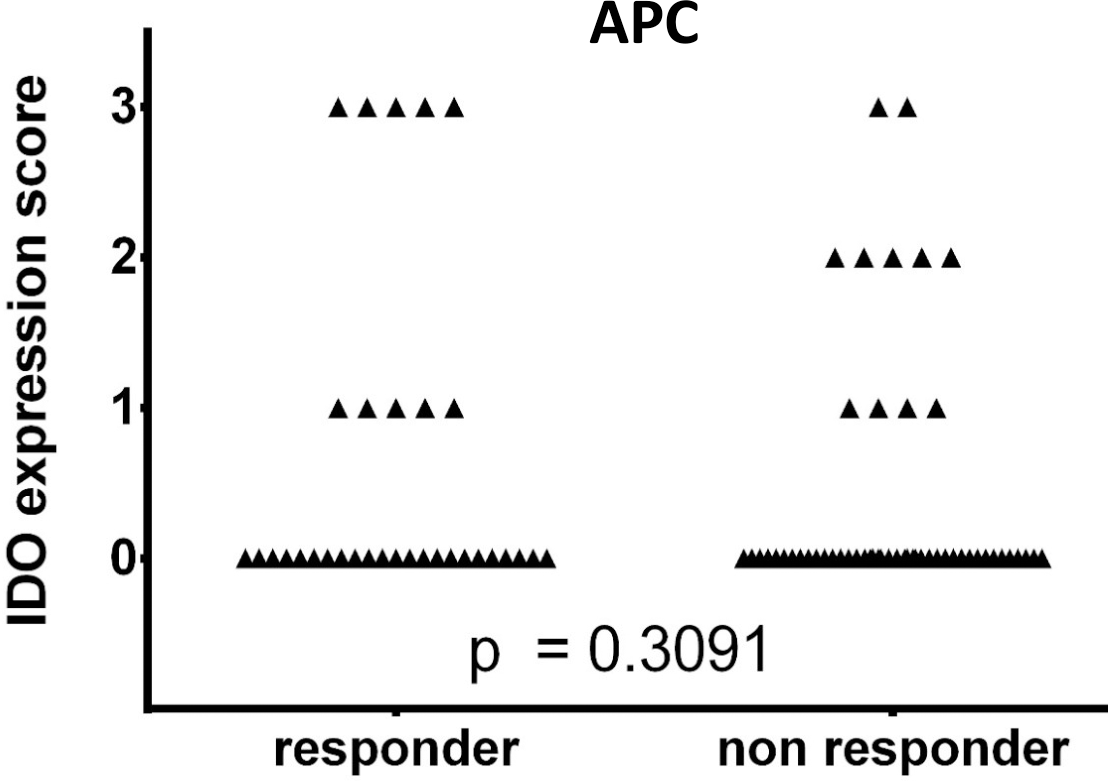
IDO expression in antigen-presenting cells in responders and non-responders to immune checkpoint inhibitor therapy. Investigation in antigen-presenting cells (APCs) for patients with therapy benefit (CR, PR or SD), so-called ‘responders’ (n = 33), and non-responding patients (PD) (n = 53) to therapy with ipilimumab. PD: progressive disease; SD: stable disease; PR: partial response; CR: complete response.

### Data analyses

Expression of IDO was compared between responders to checkpoint inhibitor therapy and non-responders. Patients with Complete Response (CR), Partial Response (PR), Mixed Response (MR) and Stable Disease (SD) were considered responders, while patients with Progressive Disease (PD) were included in the non-responder group. To qualify as responder, patients had to demonstrate stable disease (SD) for at least three months. All patients were staged radiologically via RECIST/immune related (IR) response criteria.

Median overall survival (mOS) was calculated using the Kaplan-Meier method as the time from diagnosis of stage IV disease until melanoma-specific or treatment-related death and disease progression, respectively. If no such event occurred or if a patient was lost to follow-up, the date of the last documented contact was registered and used as a censored observation. The log-rank test compared survival curves. Comparisons of IDO expression or PD-L1 expression and treatment response were performed with Mann-Whitney test. Two-tailed p-values were calculated and considered significant with values p < 0.05. A Spearman correlation between the IHC expression scores of IDO and PD-L1 in tumor tissue was accomplished. Analyses were performed with GraphPad Prism version 5.01 and 9.3.1 (GraphPad Software, Inc., San Diego, California, USA).

## Results

In order to evaluate the influence of PD-L1 and IDO expression on response to immunotherapy and survival, protein expression was assessed in 86 pretreatment melanoma biopsies from patients with metastatic disease before initiation of checkpoint inhibitor therapy. A total of 64 patients was assessed in a group enriched for responders. Patient characteristics are shown in Table 1.

**Table 1:** Patient characteristics and sites of tumor biopsies.

Patient characteristics	Total	Clinical benefit	No clinical benefit
Age	61 years (29–86)	57.6	60.8
Gender	29% female/71% male	36% female	25% female
Number of specimens	86	33 (38%)	53 (62%)
Number of pretreatments	3 (0–6)	3	3
**Response**			
PD	53		53
SD	20	20	
PR	11	11	
CR	2	2	
**Origin of tissue**			
Primary tumor	2	1	1
Cutaneous metastases	42	17	25
Nodal metastases	24	6	18
Visceral metastases	14	7	7
Brain metastases	2	2	0
Other or not specified	2	1	1

Clinical benefit was defined as CR, PR or SD for at least 3 months. PD: Progressive disease; SD: Stable disease; PR: Partial response; CR: Complete response. Number of pretreatments: rounded*.*

Patients had been heavily pretreated before undergoing checkpoint inhibitor therapy with more than 48% receiving at least three prior treatment regimens (Fig 1). More than 61% of the patients received further treatment after therapy with ipilimumab. This percentage did not differ between responders and non-responders.

In total, 33 specimens of 23 responders and 53 specimens of 41 non-responders to ipilimumab therapy were analyzed for IDO expression (Fig 2C). Out of these specimens, 24 were from nodal involvement (either sentinel or recurrent disease), and 60 specimens were non-nodal metastases with 2 primary tumors. The median tumor content was 60%. Sites of tumor probes are given in Table 1. In each slide, the IDO expression was evaluated separately for the tumor and the antigen presenting cells (APC). In 78% (n = 67) of the specimens, IDO expression was consistent between tumor and APC, whereas 22% (n = 19) of specimen showed IDO expression either in the tumor or the APC. In 58% (n = 50) of the specimens, no IDO expression could be detected.

### Response to checkpoint inhibitor therapy

Clinical benefit, defined as either SD, PR, or CR for at least 3 months, corresponded to 38% (n = 33) of the specimens (Table 1). Regardless whether IDO expression was observed in APCs or tumor, responders were considered IDO-positive in 42% (n = 14) of the cases. Similarly, in the non-responder specimen, 42% (n = 22) were considered IDO-positive. There was no statistically significant correlation between overall IDO expression and response to ipilimumab, also when dividing the specimens into IDO expression in the tumor cells (Fig 2A) or in the APCs (Fig 3).

Similarly, there was no difference in response with regard to PD-L1 expression in tumor cells (Fig 2B).

### No correlation between IDO and PD-L1 expression

**Fig 4:**
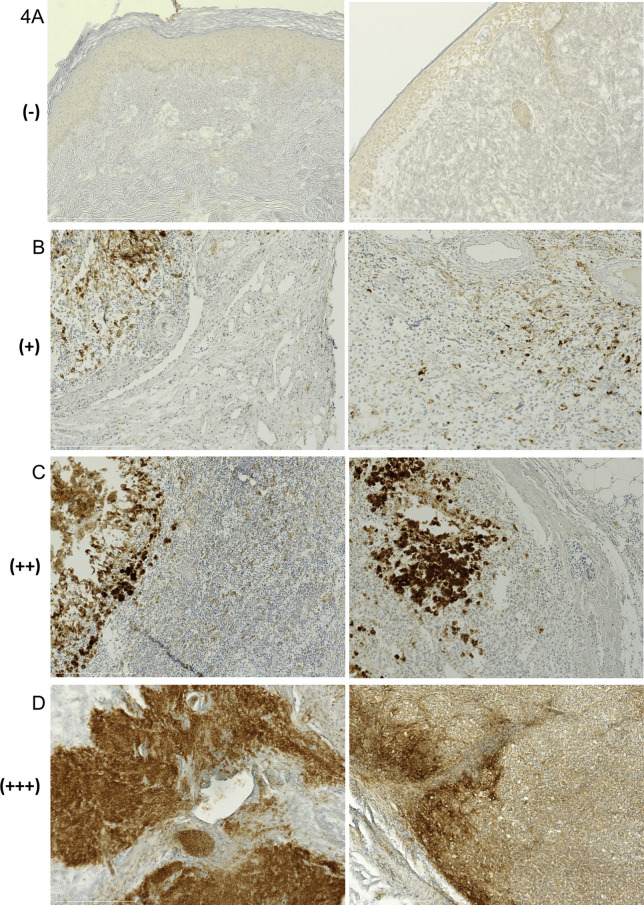
IHC scoring. Staining against PD-L1 (clone E1L3N), demonstration of IHC scoring, examples for score values (- to +++). (A) Score intensity (-) was defined as IHC-negative, two separate tissue samples. (B) Intensity value (+) defined as < 25% of IHC-positive cells, two different regions of one sample. (C) Score (++) included 25-50%, two regions of one sample. (D) Intensity (+++) was defined as > 50% of IHC-positive cells, different regions of one sample. Nodal (B, C) and non-nodal (A, D) tissue specimen. Hundredfold magnification.

**Fig 5:**
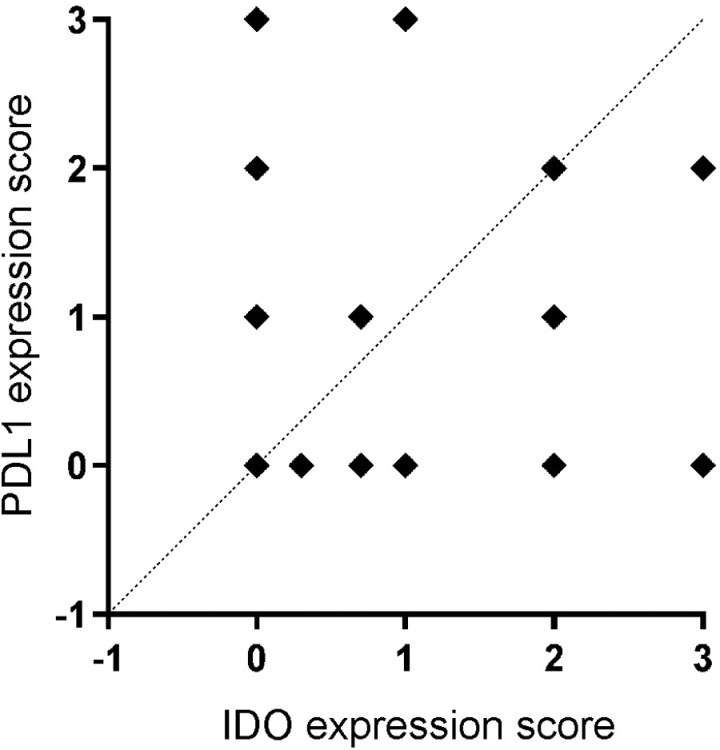
Correlation of immunohistochemical expression scores of PD-L1 and IDO in tumor cells. Spearman correlation; p = 0.6409; n = 86.

Next, we assessed whether IDO expression was correlated with PD-L1 expression. Blinded reviewers evaluated PD-L1 expression from (+) to (+++) (Fig 4A–D) in tumor biopsies (Fig 2D). Spearman correlation between the expression rates of IDO and PD-L1 in tumor tissue revealed no significant correlation between IDO and PD-L1 expression in baseline tumor biopsies (Fig 5, p = 0.6409).

### Survival rates

**Fig 6:**
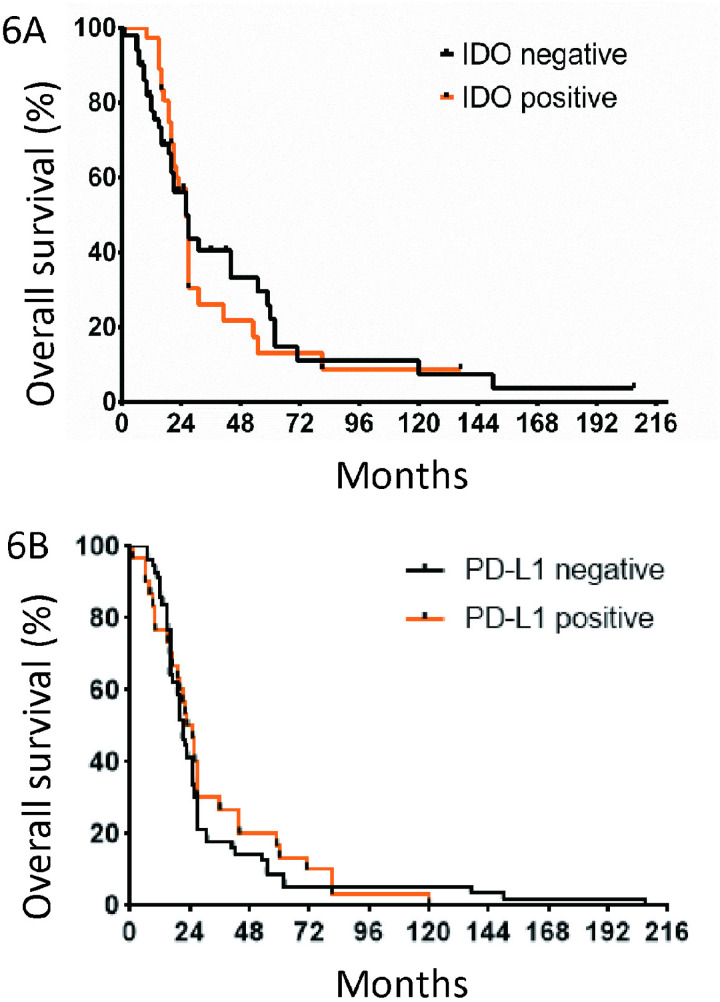
Overall survival related to IDO and PD-L1 expression. (A) Median overall survival of patients with metastatic melanoma with positive compared to negative IDO expression in pretreatment tumor biopsies (n = 86) (tumor cells or APC). APC: antigen presenting cells. (B) Median overall survival of patients with metastatic melanoma with PD-L1-positive and PD-L1-negative staining of tumor biopsies.

To analyze its role as a prognostic marker we performed survival analyses of patients with regard to IDO expression. The mOS was 26 months in IDO-positive and IDO-negative patients regardless of staining in tumor or APCs (Fig 6; p = 0.9501).

**Fig 7:**
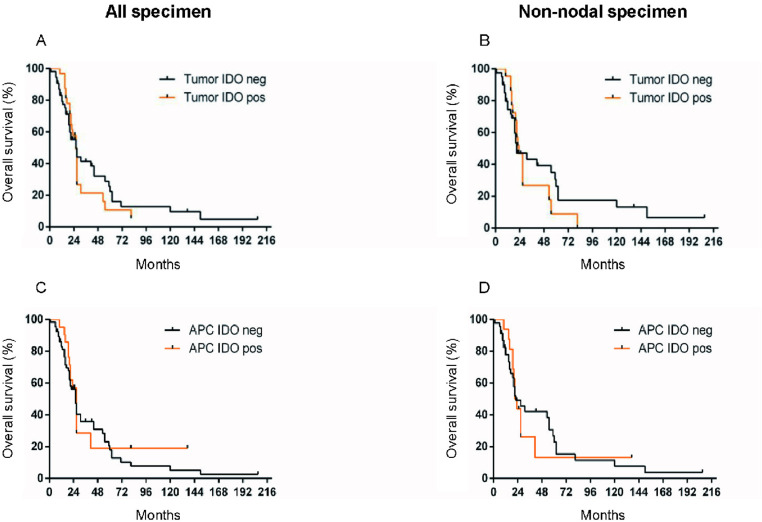
Overall survival in all vs. non-nodal specimen related to IDO expression in tumor cells or APCs. Median overall survival of patients with metastatic melanoma with IDO-positive compared to IDO-negative staining of pretreatment biopsies (n = 86) in tumor cells (A, B) or APC (C, D) in all specimen (A, C) or non-nodal specimen only (B, D). APCs: antigen presenting cells.

In the tumor only (without APCs), the mOS was 26 months for IDO-negative and IDO-positive specimens (Fig 7A; p = 0.6920). When assessing the non-nodal tumor specimens, the mOS was 23 months in IDO-positive and 21 months in IDO-negative biopsies (Fig 7B; p = 0.5349). The mOS of all specimens comparing IDO expression on the APCs only was 27 months in IDO-positive specimens vs. 26 months in IDO negative specimens (Fig 7C; p = 0.5675). In non-nodal specimens IDO expression assessed on the APCs, the specimens with positive IDO expression had a mOS of 22 months vs. 22 months in IDO-negative specimens (Fig 7D; p = 0.8702).

For PD-L1 expression, the mOS was 24 months in PD-L1-positive and 21 months for PD-L1-negative patients (Fig 6B; p = 0.6322).

## Discussion

This study investigated whether IDO expression in biopsies of melanoma metastases taken before initiation of checkpoint inhibitor therapy is predictive for response to ipilimumab. In addition, the correlation of IDO and PD-L1 expression was investigated as well as the correlation of IDO and PD-L1 expression to overall survival. To our knowledge, this is the first described cohort investigating the IDO-dependent response to ipilimumab in melanoma in the context of PD-L1 expression. Previously, single reports have suggested potential biomarkers to be possibly associated with clinical activity, like forkhead box P3 and IDO, a post-treatment increase in tumor-infiltrating lymphocytes [50] and expression of immune-related genes like TH1 associated cytokines (CCL4, CCL5, CXCL9, 10) as well as markers for CD8 + cytotoxic T cells (i.e., Perforin1 and Granzyme B) [50,51].

Studies on the tumor microenvironment (TME) demonstrate different mechanisms involved in immunosuppression in melanoma tumors: CD8 + T cell migration depends on the elevated expression of, i.e., IDO, but also FoxP3 or PD-L1 [22]. Murine studies additionally suggest that PD-L1 (similar to IDO and Treg) might follow CD8 + T cell infiltration as a negative feedback loop mediated by interferon-γ [22]. Thus, cancer therapy targeting negative regulatory immune checkpoints might be preferentially beneficial in patients with a T-cell rich TME [2,22]. Furthermore, we are aware of the TME’s complexity and we therefore make no claim to completeness.

In contrast to previous reports [19,52–57], we observed no correlation of IDO expression and survival in our melanoma patient cohort treated with ipilimumab. As shown before, we did not examine a correlation of PD-L1 expression and survival [58]. However, some of these patients analyzed previously did not receive subsequent immunotherapy [52]. Moreover, our cohort consists of mostly non-nodal tumors which is closer to the real-life situation where ipilimumab is used for stage IV (AJCC) disease, whereas previous reports mainly focused on nodal specimen or primary tumors [19,52–54,59]. Additionally, prior treatment – as was the case in our patient cohort - may influence the IDO expression [57]. As described previously, immunotherapy hardly influences PD-L1 or IDO expression over time, whereas targeted therapy might lead to an increased PD-L1 and IDO expression [24,57]. In the evaluation of PD-L1 expression in melanoma, these factors as well as different results depending on the antibodies used [60] may be the reason for controversial results [61]. Even though for IDO, the 10.1 clone is widely in use [25], for PD-L1 different clones are used, i.e., E1L3N, clone 28–8 or 5H1, whereas SP142 has shown to be less sensitive [62–64]. Therefore, future studies should not include SP142. Furthermore, evaluation based on mRNA levels as previously reported for PD-L1 in melanoma [60] may be more reliable for investigating clinical correlations with biomarkers. In renal cell carcinoma [65,66] and breast cancer [67], high IDO expression assessed via immunohistochemistry and as mRNA by RT-PCR was correlated with an increased overall survival.

The observed lack of correlation of IDO and PD-L1 expression could be due to differential longitudinal expression. IDO expression in tumor negative sentinel lymph nodes confers a negative prognostic value for patients [19]. Thus, IDO expression could be a very early determinant of immune instruction in melanoma development whereas PD-L1 expression would rather express an exhausted immune system at the later end of cancer development.

Additionally, other reasons for the lacking correlation could be distinct regulatory pathways, as IDO might be indirectly influenced by hypoxia, e.g., through IFN-gamma, whereas PD-L1 can be upregulated by hypoxia by direct transcriptional regulation which is not known for IDO [68]. Other factors might be TME heterogeneity depending on different tumor regions and tumor types, epigenetic modifications and pretreatments which might independently influence IDO or PD-L1 expression.

IDO plays a role in several cancer entities and is associated with a negative outcome [69]. Although we did not show a correlation of IDO expression to overall survival, this might be in accordance with previous discouraging results of anti-IDO regimes in a phase 3 study [38]. Even if the combined use of anti-IDO and anti-PD1 is much debated, the outcome and its clinical relevance remains unclear so far [70]. The anti-IDO vaccine in combination with anti-PD-L1 vaccine, with synergistic effects in generating cytotoxic T cells, is currently being tested in a larger clinical trial (NCT03047928) [70]. We think our results are close to reality since we also included non-nodal metastases. Nevertheless, the dynamic interplay of IDO, PD-L1 and the immune system in melanoma remains to be further investigated.

Other pathophysiological processes such as autoimmune diseases, infections, allergic and cardiovascular disorders, as well as depression have also been linked to the IDO dependent catabolism of tryptophan to kynurenine and its downstream metabolites [67,71]. Metabolism has been shown to interact with the gut microbiome which in turn is linked to response and survival after checkpoint inhibitor therapy [41,42,72].

Taking all data into account, IDO seems to be a highly relevant molecule in the antitumor response. However, its expression patterns in tumor cells and APCs are difficult to employ as predictive biomarkers for checkpoint inhibitor therapy.

## Supporting information

S1 Table Clinical data. Raw data provided.
(XLSX)
